# Changes in the Adult GluN2B Associated Proteome following Adolescent Intermittent Ethanol Exposure

**DOI:** 10.1371/journal.pone.0155951

**Published:** 2016-05-23

**Authors:** H. Scott Swartzwelder, Mary-Louise Risher, Kelsey M. Miller, Roger J. Colbran, Danny G. Winder, Tiffany A. Wills

**Affiliations:** 1 Durham VA Medical Center, Duke University Medical Center, Durham, North Carolina, United States of America; 2 Department of Psychiatry and Behavioral Sciences, Duke University Medical Center, Durham, North Carolina, United States of America; 3 Department of Psychology and Neuroscience, Duke University Medical Center, Durham, North Carolina, United States of America; 4 Department of Molecular Physiology & Biophysics, Vanderbilt University, Nashville, TN, United States of America; 5 Vanderbilt Brain Institute, Vanderbilt University, Nashville, TN, United States of America; 6 J. F. Kennedy Center for Research on Human Development, Vanderbilt University, Nashville, TN, United States of America; 7 Department of Cell Biology and Anatomy, Louisiana State University Health Science Center, New Orleans, LA, United States of America; University of Victoria, CANADA

## Abstract

Adolescent alcohol use is the strongest predictor for alcohol use disorders. In rodents, adolescents have distinct responses to acute ethanol, and prolonged alcohol exposure during adolescence can maintain these phenotypes into adulthood. One brain region that is particularly sensitive to the effects of both acute and chronic ethanol exposure is the hippocampus. Adolescent intermittent ethanol exposure (AIE) produces long lasting changes in hippocampal synaptic plasticity and dendritic morphology, as well as in the susceptibility to acute ethanol-induced spatial memory impairment. Given the pattern of changes in hippocampal structure and function, one potential target for these effects is the ethanol sensitive GluN2B subunit of the NMDA receptor, which is known to be involved in synaptic plasticity and dendritic morphology. Thus we sought to determine if there were persistent changes in hippocampal GluN2B signaling cascades following AIE. We employed a previously validated GluN2B-targeted proteomic strategy that was used to identify novel signaling mechanisms altered by chronic ethanol exposure in the adult hippocampus. We collected adult hippocampal tissue (P70) from rats that had been given 2 weeks of AIE from P30-45. Tissue extracts were fractionated into synaptic and non-synaptic pools, immuno-precipitated for GluN2B, and then analyzed using proteomic methods. We detected a large number of proteins associated with GluN2B. AIE produced significant changes in the association of many proteins with GluN2B in both synaptic and non-synaptic fractions. Intriguingly the number of proteins changed in the non-synaptic fraction was double that found in the synaptic fraction. Some of these proteins include those involved in glutamate signaling cytoskeleton rearrangement, calcium signaling, and plasticity. Disruptions in these pathways may contribute to the persistent cellular and behavioral changes found in the adult hippocampus following AIE. Further, the robust change in non-synaptic proteins suggests that AIE may prime this signaling pathway for future ethanol exposures in adulthood.

## Introduction

Alcohol use is generally initiated during adolescence and is often consumed in a ‘binge like’ manner by adolescents and young adults. While adolescent alcohol use is relatively common, it is not without its deleterious consequences. This is most importantly demonstrated by the fact that alcohol use during adolescence is known to be the largest predictor for future alcoholism [1]. It is therefore important to understand what neural adaptations occur during and after adolescent alcohol exposure and how they might contribute to future dependence and other neuropsychological outcomes.

Rodent models have contributed heavily to the emerging understanding of the neural and behavioral consequences of adolescent alcohol exposure. For example, adolescent rodents are known to be more sensitive than adults to the memory impairing effects of ethanol in a hippocampal dependent task. Further, this enhanced sensitivity to memory impairment appears to become “locked in” with chronic adolescent ethanol exposure [2]. Later studies found this “locked in” phenotype of memory impairment in adulthood was associated with a significant increase in immature dendritic spines and alterations in the threshold for hippocampal synaptic plasticity induction [3]. Interestingly, each of these phenotypes is related to NMDAR function.

During adolescence there is an overall decrease in excitatory tone, pruning of glutamatergic synapses, and shifts of glutamate receptor (NMDAR) subunit composition [4–9]. In the hippocampus, NMDARs transition from those containing primarily GluN2B subunits to those containing GluN2A [8]. Importantly, NMDARs are major targets for the effects of ethanol and may thereby mediate some of the differences in ethanol sensitivity. In fact, ethanol has been shown to inhibit NMDAR function more potently in the adolescent hippocampus compared to adults [10] and NMDAR-dependent long-term potentiation is disrupted more by ethanol exposure in adolescents than adults [11, 12]. Thus, the NMDAR subunit shift that occurs during adolescence may be responsible for theses differences, since subunit composition confers many of receptor properties like decay time and associated signaling cascades[13, 14].

There has been a substantial amount of work in adult animals evaluating the subunit selectivity of ethanol’s effects, which have yielded mixed results (reviewed in [15]). However, a recent study that combined genetics with pharmacological antagonism finds that the acute inhibitory effects of ethanol are GluN2B specific in the bed nucleus of the stria terminalis [16]. In response to chronic ethanol administrations and withdrawal, the GluN2B containing NMDARs are consistently increased [16–25]. Further, during withdrawal GluN2B-NMDARs appear to relocate to extrasynaptic sites [16, 26]. This enhancement of extrasynaptic GluN2B-NMDAR signaling could be a potential mechanism underlying the effects of AIE in the hippocampus.

A proteomic approach was recently used to evaluate changes in GluN2B-NMDAR signaling from synaptic and non-synaptic sites during withdrawal from chronic ethanol in adults [27]. Due to the successful application of this approach to study the effects of chronic ethanol in adulthood, we used a GluN2B specific proteomic approach to assess the effects of chronic ethanol exposure during adolescence on the adult hippocampus. We were able to identify a number of proteins that were altered by AIE in both synaptic and non-synaptic fractions. These proteins are involved in many of the signaling pathways associated with the phenotypes that are altered by AIE.

## Materials and Methods

### Animals/Adolescent Intermittent Ethanol Exposure

The experiments in this study were conducted in accordance with guidelines from the American Association for the Accreditation of Laboratory Animal Care and the National Research Council’s Guide for Care and Use of Laboratory Animals. These procedure were also approved by the Durham VA Medical Center, the Duke University Animal Care and Use Committees, and Vanderbilt University Animal Care and Use Committee. All animal procedures were conducted under active protocols, approved and monitored by both the Duke University Medical Center and Durham VA Medical Center Institutional Animal Care and Use committees (IACUCs). Those protocols included specific endpoints for any rats that became seriously ill or moribund at any point prior to the termination of the study. During the treatment phase of the study all animals were monitored for physical condition once daily by research staff and once daily by Durham VA Medical Center Veterinary staff. The criteria used to make those health and welfare assessments were in place at the Durham VA medical Center vivarium as applied to all animal studies there, with additional oversight by the Duke University Medical Center Animal Care and Use Program. The assessments included both behavioral and physiological monitoring under direct Veterinarian supervision.

**Fig 1:**
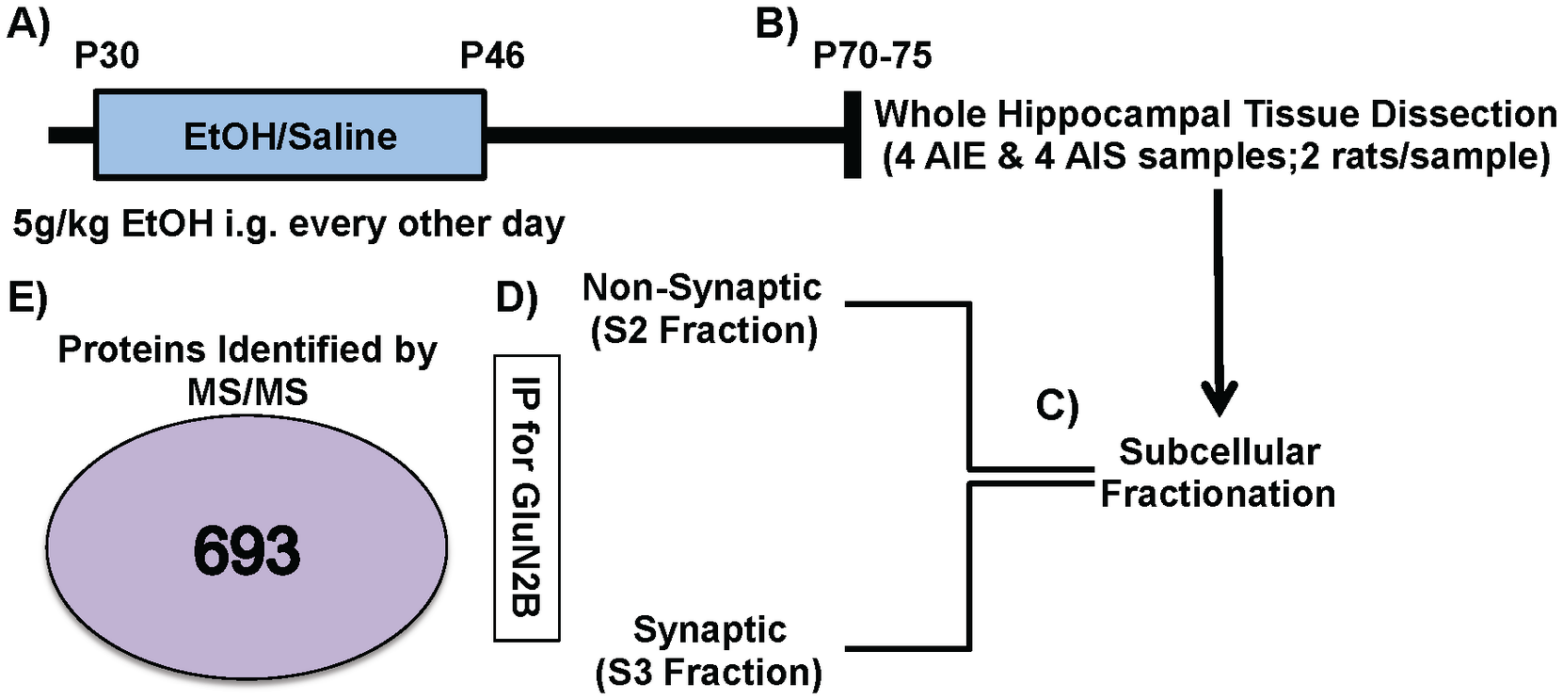
Schematic of Proteomic Analysis. (A) Adolescent Intermittent Ethanol (AIE) or Saline (AIS) Exposure: Rats received intragastric (i.g.) 5g/kg ethanol or saline every other day from postnatal day (P) 30–46. (B) The hippocampus was dissected from brains collected in adulthood between P70-75 (8 AIE & 8 AIS; two rats/sample). (C) Subcellular Fractionation. Synaptic-enriched/S3 [triton- & deoxycholate (DOC)-soluble] and non-synaptic/S2 (triton-soluble) fractions were generated. (D) GluN2B Immunoprecipitation (IP) was performed on S2 and S3 fractions from AIE and AIS treatments. (E) Mass spectrometry identified 696 proteins across all samples.

Male PD 25 Sprague-Dawley rats (Charles River, USA) were double housed and maintained in a temperature and humidity controlled room with *ad libitum* access to food and water at Duke University Medical Center and Durham VA Medical Center. Animals were dosed as was described previously in [3, 28]. Briefly, animals were allowed to acclimatize for 5 days in a vivarium on a reverse 12:12-hr light:dark cycle (lights off at 9:00 am) prior to beginning adolescent intermittent ethanol (AIE) or adolescent intermittent saline (AIS) administration. All animals were exposed to AIE or AIS beginning PD 30 and received either 10 doses of 5 g/kg ethanol (35% v/v in saline at 18.12 mL/kg) or isovolumetric saline through intragastric gavage. This treatment was administered using a 2 days on, 1 day off, 2 days on, 2 days off intermittent schedule for 16 days (PD 30–46). This AIE treatment has been shown to produce blood ethanol concentrations (BEC) in the range of 172–199 mg/dl [3]. Rats were then allowed to mature to adulthood (PD 70–75) and then anesthetized with isoflurane and decapitated for tissue collection (see Fig 1). The whole hippocampus was dissected from AIE and AIS treated rats. The hippocampal tissues from two rats were combined into each sample (4 samples/treatment). This was done to achieve optimal protein concentrations and reduce individual variations between individual rat treatments.

### Subcellular Fractionation & GluN2B Immunoprecipitations

Synaptic (triton & deoxycholate (DOC)-soluble) and non-synaptic (triton-soluble) fractions were generated from all of these tissue samples [27, 29, 30] see Fig 1C. Tissue was homogenized in homogenization buffer (150 mM KCl, 50 mM Tris—HCl pH 7.5, 1 mM DTT, 0.2 mM PMSF, 1 mM benzamidine, 1 μM pepstatin, 10 μg/ml leupeptin, and 1 μM microcystin-LR) using Kontes glass tissue grinders at 4°C. Total homogenate was rocked for 30 min at 4°C and spun down at 100,000 x g for 1 h in Type 70.1 rotator yielding an S1 fraction (soluble cytosolic protein pool) and a P1 pellet (insoluble fraction). The cytosolic fractions (S1) were not used in further analysis. P1 was re-suspended in homogenization buffer containing 1% (v/v) Triton X-100 using a Kontes, rounded tip cone pestle and rocked for 30 min at 4°C. The homogenate was then spun down at 16,000 x g for 10 min at 4°C yielding an S2 fraction (membrane-associated protein pool) and a P2 pellet (Triton-insoluble fraction). The P2 was sonicated at 4°C in Homogenization buffer containing 1% (v/v) Triton X-100 and 1% (w/v) sodium deoxycholate and rocked for 30 min at 4°C yielding the S3 fraction (membrane-associated protein pool). Precleared samples (300 μ) were incubated at 4°C for 1 h with mouse antisera to GluN2B (7.5 μl; antibody from BD Transduction Laboratories), protein G magnetic beads (Pierce Biotechnology) were added, and samples were incubated overnight at 4°C. Immunoprecipitates were washed 3 times with 1 ml of immunoprecipitated buffer (50 mM Tris-HCl, pH 7.5, 150 mM sodium chloride, 0.5% Triton X-100) and suspended in 2 x SDS sample buffer and separated by SDS-PAGE [27, 29, 31] see Fig 1D.

### Mass Spectrometry

Coomassie stained blots of GluN2B IPs sample lanes were subdivided into three separate samples to maximize protein coverage in the mass spectrometer (MS). Co-IPs from the synaptic and non-synaptic fractions in AIE and AIS rats were then analyzed via LC-MS/MS (Vanderbilt Proteomics Core; LTQ-Orbitrap Velos). MS/MS spectra of the peptides were obtained using data-dependent scanning, in which one full MS spectra was followed by sixteen MS/MS spectra, drawn from the most intense ions observed in each MS spectra. Peptiome 1.1.50 identified peptides corresponding to the MS/MS scans by comparing them to spectra from the NIST rat ion trap library or an equal number of shuffled decoy spectra. Scaffold3 (V3.6.4) was used to filter raw identifications, conduct protein assembly, and produce tables of spectrum counts. A peptide-spectrum match with a false discovery rate of 5% was applied, as well as a two-distinct-peptide-sequence-per-protein identification filter. Identified contaminants from wool, cotton, and saliva, as well as sequences not present in both normal and reversed orientations were removed.

### Proteomic Statistics

Fisher’s exact test was used to test if the spectral counts were different between experiment/control groups. A 2 by 2 contingency table is constructed for each protein. The 4 components in the table were A) spectral counts for this protein in the experimental group, B) spectral counts for this protein in the control group, C) spectral counts for all other proteins in the experimental group, and D) spectral counts for all other proteins in the control group. These analyses were performed at Louisiana State University Health Science Center.

Ingenuity pathway analysis (IPA) was also used for proteins that were significantly changed by AIE in the synaptic and non-synaptic fraction.

## Results

### Adolescent Intermittent Ethanol-Induced Changes in the GluN2B Proteome in Adulthood

In the current set of studies, we employed the use of a discovery-based proteomic approach to elucidate alcohol-induced changes in the GluN2B-NMDA receptor proteome. To do this, we exposed adolescent rats to either intermittent ethanol (AIE) or intermittent saline (AIS) and collected hippocampal tissue in adulthood. This tissue was then fractionated to separately enrich synaptic and non-synaptic proteins, immunoprecipitated (IP) using an antibody against GluN2B, and proteins were separated by SDS-PAGE. Gel lanes were excised from the gels, and the proteins digested using trypsin. Digests were then analyzed by a mass spectrometry (MS, LTQ-Orbitrap). This proteomic analysis identified 693 total proteins (Fig 1E) with 586 detected in the synaptic fraction and 304 detected in the non-synaptic fraction (for complete list see S1 Dataset). Of these GluN2B associated proteins, 34 were significantly different between AIE and AIS groups (see Table 1). A majority (85%) of these proteins were increased in their association with GluN2B after AIE. In the non-synaptic fraction, there were 73 proteins that were significantly different between AIE and AIS groups (see Table 2). In this fraction, a majority (74%) of proteins also displayed increased fold changes in the AIE group. Spectral counts for the GluN2B subunit were not significantly different between AIE and AIS in either fraction (S2: p = 0.47; S3: p = 0.08).

**Table 1:** Proteins changed by adolescent intermittent ethanol exposure (AIE) in S3 (synaptic) fraction.

Protein	Accession#	AIS spectral counts	AIE spectral counts	Fold Δ	P value
Glutamate [NMDA] receptor subunit 2B (GRIN2b, GluN2B)	G3V746	375	372	NA	N.S.
Glutamate [NMDA] receptor subunit zeta-1 (GRIN1, GluN1)	P35439	268	339	NA	N.S.
Disks large homolog 4 (DLG4, PSD-95)	P31016	656	668	NA	N.S.
2-oxoglutarate dehydrogenase, mitochondrial (OGDH)	Q5XI78	5	20	4.00	0.01
2',3'-cyclic-nucleotide 3' phosphodiesterase (CNP)	P13233	65	122	1.88	0.00
6-phosphofructokinase (PFKM)	Q52KS1	9	26	2.89	0.01
6-phosphofructokinase type C (PFKP)	P47860	3	12	4.00	0.04
Actin-binding LIM protein family, member 2	D3ZB53	19	38	2.00	0.04
Actin, cytoplasmic 1 (ACTB)	P60711	850	782	-1.09	< 0.0001
Alpha-actinin-1 (ACTN1)	Q6GMN8	260	373	1.43	0.00
Alpha-actinin-4 (ACTN4)	Q9QXQ0	156	220	1.41	0.03
AP-2 complex subunit alpha-2 (AP2A2)	P18484	98	144	1.47	0.04
Calcium/calmodulin-dependent protein kinase II, beta (CAMK2B)	G3V9G3	534	469	-1.14	< 0.0001
Erythrocyte protein band 4.1-like 3 (EPB41L3)	Q9JMB3	44	72	1.64	0.05
Fascin (FSCN1)	P85845	0	13	13.00	0.00
Heat shock cognate 71 kDa protein (HSPA8)	P63018	100	49	-2.04	< 0.0001
Muscular LMNA-interacting protein (MLIP)	Q569A0	11	33	3.00	0.00
Matrin-3 (MRT3)	P43244	20	41	2.05	0.03
Neural cell adhesion molecule 1 (NCAM1)	P13596	16	42	2.63	0.00
Bassoon (BSN)	G3V984	41	68	1.66	0.05
Alpha-actinin-2 (ACTN2)	D3ZCV0	297	442	1.49	0.00
Spectrin alpha chain, brain (SPTAN1)	P16086	1523	1854	1.22	0.02
Spectrin beta 1 (SPTBN1)	G3V6S0	980	1235	1.26	0.02
Transitional endoplasmic reticulum ATPase (VCP)	P46462	13	29	2.23	0.04
Tubulin beta-2A chain (TUBB2A)	P85108	1657	1715	1.04	0.01
Tubulin beta-2B chain (TUBB2B)	Q3KRE8	1573	1653	1.05	0.05
Ankyrin-2 (ANK2)	F1M9N9	466	611	1.31	0.01
Agrin (ARGN)	F1LQ53	8	25	3.13	0.01
Dynamin-1 (DNM1)	D4AAP9	132	198	1.50	0.01
Myosin 11 (MYH11)	F1M7T3	208	280	1.35	0.05
Neurofilament heavy polypeptide (NEFH)	F1LRZ7	739	672	-1.10	< 0.0001
Plectin (PLEC)	F1MAL6	1298	1688	1.30	< 0.0001
Myotubularin-related protein 5 (SBF1)	D3ZNN0	10	24	2.40	0.04
Spectrin alpha chain (SPTAN1)	E9PSZ3	1078	1704	1.58	< 0.0001
Spectrin beta chain (SPTBN2)	F1MA36	752	965	1.28	0.01
V-type proton ATPase 116 kDa subunit a (ATPA6V0A1)	P25286	48	101	2.10	0.00
Vesicle-fusing ATPase (NSF)	Q9QUL6	167	135	-1.24	0.00

Proteins and accession numbers were identified using NIST rat ion trap library. The next columns give the summated spectral counts for all samples (n = 4) in adolescent intermittent ethanol exposure (AIE) or adolescent intermittent saline exposure (AIS) treated rats. These are followed by fold changes and p-values for treatment effects.

**Table 2:** Proteins changed by adolescent intermittent ethanol exposure (AIE) in S2 (non-synaptic) fraction.

Protein	Accession#	AIS spectral counts	AIE spectral counts	Fold Δ	P value
Glutamate [NMDA] receptor subunit 2B (GRIN2b, GluN2B)	G3V746	530	508	NA	N.S.
Glutamate [NMDA] receptor subunit zeta-1 (GRIN1, GluN1)	P35439	358	428	1.20	0.01
Disks large homolog 4 (DLG4, PSD-95)	P31016	68	94	1.38	0.04
6-phosphofructokinase (PFKM)	Q52KS1	12	28	2.33	0.01
6-phosphofructokinase	G8JLS1	10	22	2.20	0.04
Fibrinogen alpha chain (FGA)	Q7TQ70	19	60	3.16	< 0.0001
Aconitate hydratase, mitochondrial (ACO2)	Q9ER34	47	81	1.72	0.00
ADP/ATP translocase 2 (SLC25A5)	Q09073	8	0	-8.00	0.00
Arf-GAP with GTPase, ANK repeat and PH domain-containing protein 2 (AGAP2)	Q8CGU4	10	26	2.60	0.01
Aspartate aminotransferase, mitochondrial (GOT2)	P00507	13	31	2.38	0.01
ATP synthase subunit (APT5O)	Q06647	4	0	-4.00	0.04
Calnexin (CANX)	P35565	5	18	3.60	0.01
Complement C1q subcomponent subunit A (C1QA)	P31720	5	0	-5.00	0.02
Complement component 1, q subcomponent, beta polypeptide (C1QB)	G3V7N9	5	0	-5.00	0.02
Creatine kinase B-type (CKB)	P07335	9	21	2.33	0.03
Dihydrolipoyllysine-residue acetyltransferase component of pyruvate dehydrogenase complex, mitochondrial (DLAT)	P08461	31	14	-2.21	0.01
Dihydropyrimidinase-related protein 2 (DPYSL2)	P47942	14	34	2.43	0.00
Disks large homolog 4 (DLG4)	P31016	68	94	1.38	0.04
Dynamin-1 (DNM1)	P21575	59	120	2.03	< 0.0001
Endoplasmin (HSP90B1)	Q66HD0	13	28	2.15	0.02
Fibrinogen beta chain (FGB)	P14480	7	20	2.86	0.01
Fibrinogen gamma chain (FGG)	P02680	31	59	1.90	0.00
Fructose-bisphosphate aldolase A (ALDOA)	P05065	11	31	2.82	0.00
Glutamate dehydrogenase 1, mitochondrial (GLUD1)	P10860	7	23	3.29	0.00
Guanine nucleotide-binding protein G(I)/G(S)/G(T) subunit beta-1 (GNB1)	P54311	16	6	-2.67	0.03
Guanine nucleotide-binding protein G(q) subunit alpha (GNAQ)	P82471	0	5	5.00	0.03
Heat shock protein HSP 90-alpha (HSP90AA1)	P82995	36	75	2.08	0.00
Heat shock protein HSP 90-beta (HSP90AB1)	P34058	36	73	2.03	0.00
Hexokinase-1 (HK1)	P05708	37	59	1.59	0.03
Microtubule-associated protein 1A (MAP1A)	P34926	5	19	3.80	0.00
Microtubule-associated protein 6 (MAP6)	Q63560	2	17	8.50	0.00
Myelin basic protein S (Mbp)	P02688	119	79	-1.51	0.00
Plasma membrane calcium-transporting ATPase 1 (APT2B1)	P11505	49	82	1.67	0.00
Plasma membrane calcium-transporting ATPase 3 (ATP2B3)	Q64568	27	51	1.89	0.01
Plasma membrane calcium-transporting ATPase 4 (ATP2B4)	Q64542	0	30	30.00	< 0.0001
Plectin 6 (PLEC)	Q6S3A0	1	9	9.00	0.01
Potassium channel tetramerisation domain-containing 3 (KCTD3)	D3ZNX0	64	98	1.53	0.01
Proline-rich transmembrane protein 1 (Prrt1)	Q6MG82	61	39	-1.56	0.03
Piccolo (PCLO)	Q9JKS6	39	59	1.51	0.05
Pyruvate kinase isozymes M1/M2 (PKM)	P11980	14	30	2.14	0.02
Ras GTPase-activating protein SynGAP (SYNGAP1)	Q9QUH6	483	593	1.23	0.00
Bassoon (BSN)	G3V984	42	84	2.00	0.00
Tubulin beta-4B chain (TUBB4B)	G3V7C6	1443	1215	-1.19	< 0.0001
Protein Atp6v1a (APT6V1A)	D4A133	21	0	-21.00	< 0.0001
Regulating synaptic membrane exocytosis protein 1 (RIMS1)	Q9JIR4	5	15	3.00	0.03
Sarcoplasmic/endoplasmic reticulum calcium ATPase 2 (ATP2A2)	P11507	30	57	1.90	0.00
Sodium/potassium-transporting ATPase subunit alpha-1 (ATPA1)	P06685	255	382	1.50	< 0.0001
Sodium/potassium-transporting ATPase subunit alpha-2 (ATPA2)	P06686	298	433	1.45	< 0.0001
Sodium/potassium-transporting ATPase subunit alpha-3 (ATPA3)	P06687	418	564	1.35	< 0.0001
Sodium/potassium-transporting ATPase subunit alpha-4 (ATP1A4)	Q64541	114	160	1.40	0.01
Stress-70 protein, mitochondrial (HSPA9)	P48721	6	0	-6.00	0.01
Synaptic vesicle glycoprotein 2A (SV2A)	Q02563	22	40	1.82	0.02
Synaptojanin-1 (SYNJ1)	Q62910	0	4	4.00	0.05
Synaptophysin (SYP)	P07825	7	21	3.00	0.01
Tubulin alpha-1A chain (TUBA1A)	P68370	795	685	-1.16	0.00
Tubulin alpha-1B chain (Tuba1B)	Q6P9V9	860	765	-1.12	0.01
Tubulin alpha-4A chain (TUBA4A)	Q5XIF6	710	615	-1.15	0.01
Tubulin beta-2A chain (TUBB2A)	P85108	1514	1261	-1.20	< 0.0001
Tubulin beta-2B chain (TUBB2B)	Q3KRE8	1287	1077	-1.19	< 0.0001
Tubulin beta-3 chain (TUBB3)	Q4QRB4	990	831	-1.19	< 0.0001
Tubulin beta chain (TUBB)	P69897	1433	1216	-1.18	< 0.0001
Tubulin, beta 6 (TUBB6)	Q4QQV0	588	477	-1.23	0.00
Tyrosine-protein phosphatase non-receptor type substrate 1 (SIRPA)	P97710	1	7	7.00	0.03
Ubiquitin-like modifier-activating enzyme 1 (UBA1)	Q5U300	0	8	8.00	0.00
Ankyrin-2 (ANK2)	F1M9N9	7	21	3.00	0.01
Cadherin-13 (CDH13)	F1M7X3	7	1	-7.00	0.03
Cytoplasmic dynein 1 heavy chain 1 (DYNC1H1)	F1LRT9	77	105	1.36	0.04
Clathrin heavy chain 1	D4AD25	304	248	-1.23	0.01
Dual specificity protein phosphatase (DSP)	F1LMV6	42	17	-2.47	0.00
Cell cycle and apoptosis regulator protein 2 (CCAR2)	F1LM55	0	8	8.00	0.00
Spectrin alpha chain, non-erythrocytic 1 (SPTAN1)	E9PSZ3	71	110	1.55	0.00
Vacuolar protein sorting-associated protein 3 (VPS35)	G3V8A5	1	8	8.00	0.02
Uncharacterized protein	D3ZE44	110	142	1.29	0.05
V-type proton ATPase 116 kDa subunit a isoform 1 (ATP6VOA1)	P25286	114	156	1.37	0.01
Voltage-dependent calcium channel subunit alpha-2/delta-1 (CACNA2D1)	P54290	1	7	7.00	0.03

Proteins and accession numbers were identified using NIST rat ion trap library. The next columns give the summated spectral counts for all samples (n = 4) in adolescent intermittent ethanol exposure (AIE) or adolescent intermittent saline exposure (AIS) treated rats. These are followed by fold changes and p-values for treatment effects.

### Pathway Analysis of Adolescent Intermittent Ethanol-Induced Protein Changes in the Synaptic Fraction

**Fig 2:**
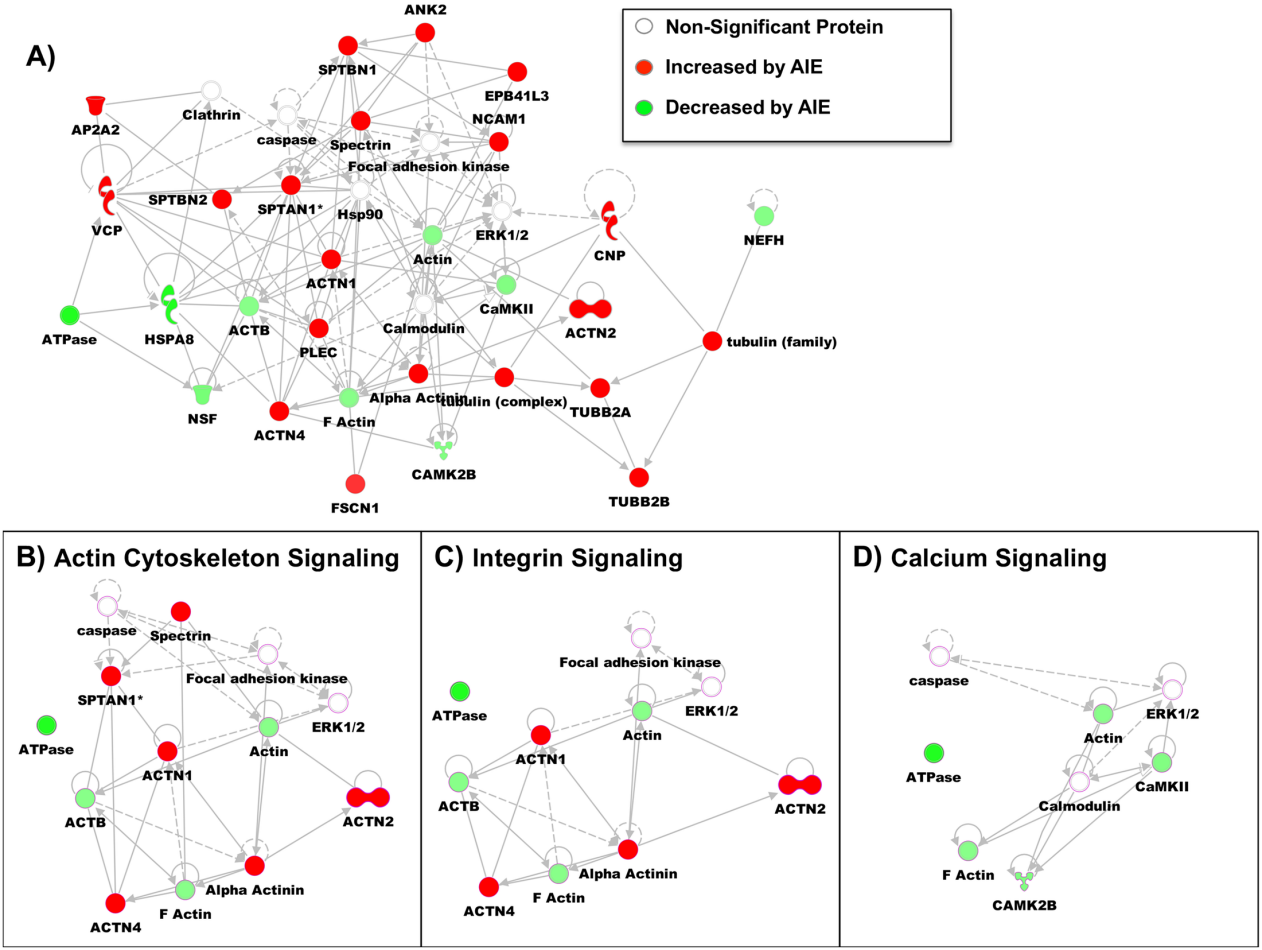
Pathway Analysis of GluN2B-associated Proteins Changed by AIE in the Synaptic Fraction. (A) Modified diagram of protein interactions identified using Ingenuity pathway analysis (IPA). Analysis was performed on significantly changed synaptic/S3 proteins by AIE (see Table 1). Color represent if the protein was increased (red) by AIE or decreased (green) by AIE. Specific signaling pathways were also identified with their protein connections: (B) Actin Cytoskeleton Signaling, (C) Integrin Signaling and, (D) Calcium Signaling.

To understand the potential function of these GluN2B-associated protein changes, the significantly changed proteins were evaluated with the Ingenuity Pathway Analysis (IPA) program. IPA identified a number of disease and functional pathways that were significantly changed by AIE in the synaptic fraction. Table 3 provides a summary of the most physiologically relevant pathways significantly changed by AIE in the synaptic fraction: cell death and survival (cell viability p = 3.10E-02, necrosis p = 1.99E-02, cell death p = 7.11E-03), cell morphology (abnormal morphology of cells p = 2.35E-03, formation of cellular protrusions p = 2.61E-08), cellular assembly and organization (organization of the cytoskeleton p = 4.65E-07), cellular compromise (degeneration of cells p = 2.71E-04), cellular growth and proliferation (proliferation of cells p = 2.49E-05), cellular movement (migration of cells p = 2.35E-02), molecular transport (transport of molecules p = 2.65E-02), neurological diseases (progressive motor neuropathy p = 2.40E-04, Huntington’s disease p = 3.89E-03, movement disorders p = 2.12E-06, dementia p = 3.00E-03, tauopathy p = 3.13E-03), and organism survival (organismal death p = 4.01E-03). In addition to these changes in specific functional pathways, the IPA program also provides a network analysis of the connectivity between significantly changed in the synaptic fraction. In this network analysis, the connectivity between database proteins is evaluated in relation to total protein interactions. The highest ranked network was able to incorporate 21 proteins (see Fig 2A). The primary functions of this network were related to cell assembly and organization, nervous system development and function, and cell morphology. A few signaling pathways from this network are highlighted in the insets: actin cytoskeleton signaling (Fig 2B), integrin signaling (Fig 2C), and calcium signaling (Fig 2D).

**Table 3:** Ingenuity Pathway Analysis of Significantly Changed S3 Proteins by AIE.

Categories	Diseases or Function	p-Value	Predicted Activation	Molecules	#
Cell Death and Survival	cell viability	3.10E-02		CAMK2B, NCAM1, NEFH, PFKM,SBF1, VCP	6
Cell Death and Survival	necrosis	1.99E-02		ACTB, AP2A2, CNP, HSPA8, NCAM1, NEFH, PFKM, PLEC,SPTBN1, VCP	10
Cell Death and Survival	cell death	7.11E-03		ACTB, ACTN4, AP2A2,CNP, HSPA8,NCAM1, NEFH, NSF,OGDH, PFKM, PLEC, SPTBN1, VCP	13
Cell Morphology	abnormal morphology of cells	2.35E-03		ACTN4, EPB41L3, NCAM1, NEFH,PFKM, PLEC, SBF1, SPTBN1	8
Cell Morphology	formation of cellular protrusions	2.61E-08	Increased	ACTB, ACTN2, ACTN4, BSN, CNP,EPB41L3, FSCN1, NCAM1,NEFH, PLEC, SPTBN1, SPTBN2	12
Cellular Assembly and Organization	organization of cytoskeleton	4.65E-07	Increased	ACTB, ACTN2, ACTN4, BSN, CNP, EPB41L3, FSCN1, NCAM1,NEFH, PLEC, SPTAN1, SPTBN1,SPTBN2	13
Cellular Compromise	degeneration of cells	2.71E-04		CNP, NCAM1, NEFH, PFKM, PLEC	5
Cellular Growth and Proliferation	proliferation of cells	2.49E-05		ACTB, ACTN1, ACTN4, ANK2, CNP, EPB41L3, FSCN1, HSPA8, NCAM1, NEFH, PFKM, PFKP, PLEC, SBF1, SPTAN1, SPTBN1, TUBB2A, VCP	18
Cellular Movement	migration of cells	2.35E-02		ACTB, ACTN4, CNP, FSCN1, NCAM1, PLEC, TUBB2B, VCP	8
Molecular Transport	transport of molecule	2.65E-02		ACTN2, AP2A2, CAMK2B, HSPA8, NSF, SPTBN1, SPTBN2	7
Neurological Disease	progressive motor neuropathy	2.40E-04		ACTB, CNP, NEFH, PFKP, TUBB2B, VCP	6
Neurological Disease	Huntington's Disease	3.89E-03		ACTB, ACTN2, CAMK2B, HSPA8, PFKM	5
Neurological Disease	Movement Disorders	2.12E-06		ACTB, ACTN2, CAMK2B, CNP, EPB41L3, HSPA8, NCAM1, PFKM, SPTBN2, TUBB2A, TUBB2B	11
Neurological Disease	Dementia	3.00E-03		ACTB, CAMK2B, CNP, NEFH, VCP	5
Neurological Disease	tauopathy	3.13E-03		ACTB, CAMK2B, CNP, NEFH,TUBB2A	5
Organismal Survival	organismal death	4.01E-03	Decreased	ACTB, ACTN4, CNP, EPB41L3, FSCN1, NCAM1, PFKM, PLEC,SBF1, SPTBN1, VCP	11

Ingenuity pathway analysis was performed on proteins that were significantly different between AIE and AIS (listed in Table 1) in the S3 synaptic fraction. The analysis identified the presence of these proteins (molecules column) in known pathways (categories column), the function or disease involvement of these pathways (disease or function column), and the number of proteins found in each pathway (# column). Further, p-values were generated for the identified proteins compared to the rat genome. Predicated activation of a pathway was determined using the fold change for each protein. The full protein names for the abbreviations in the molecules column can be found in Table 1.

Ingenuity pathway analysis was performed on proteins that were significantly different between AIE and AIS (listed in Table 2) in the S2 non-synaptic fraction. The analysis identified the presence of these proteins (molecules column) in known pathways (categories column), the function or disease involvement of these pathways (disease or function column), and the number of proteins found in each pathway (# column). Further, p-values were generated for the identified proteins compared to the rat genome. Predicated activation of a pathway was determined using the fold change for each protein. The full protein names for the abbreviations in the molecules column can be found in Table 2.

### Pathway Analysis of Adolescent Intermittent Ethanol-Induced Protein Changes in the Non-Synaptic Fraction

**Fig 3:**
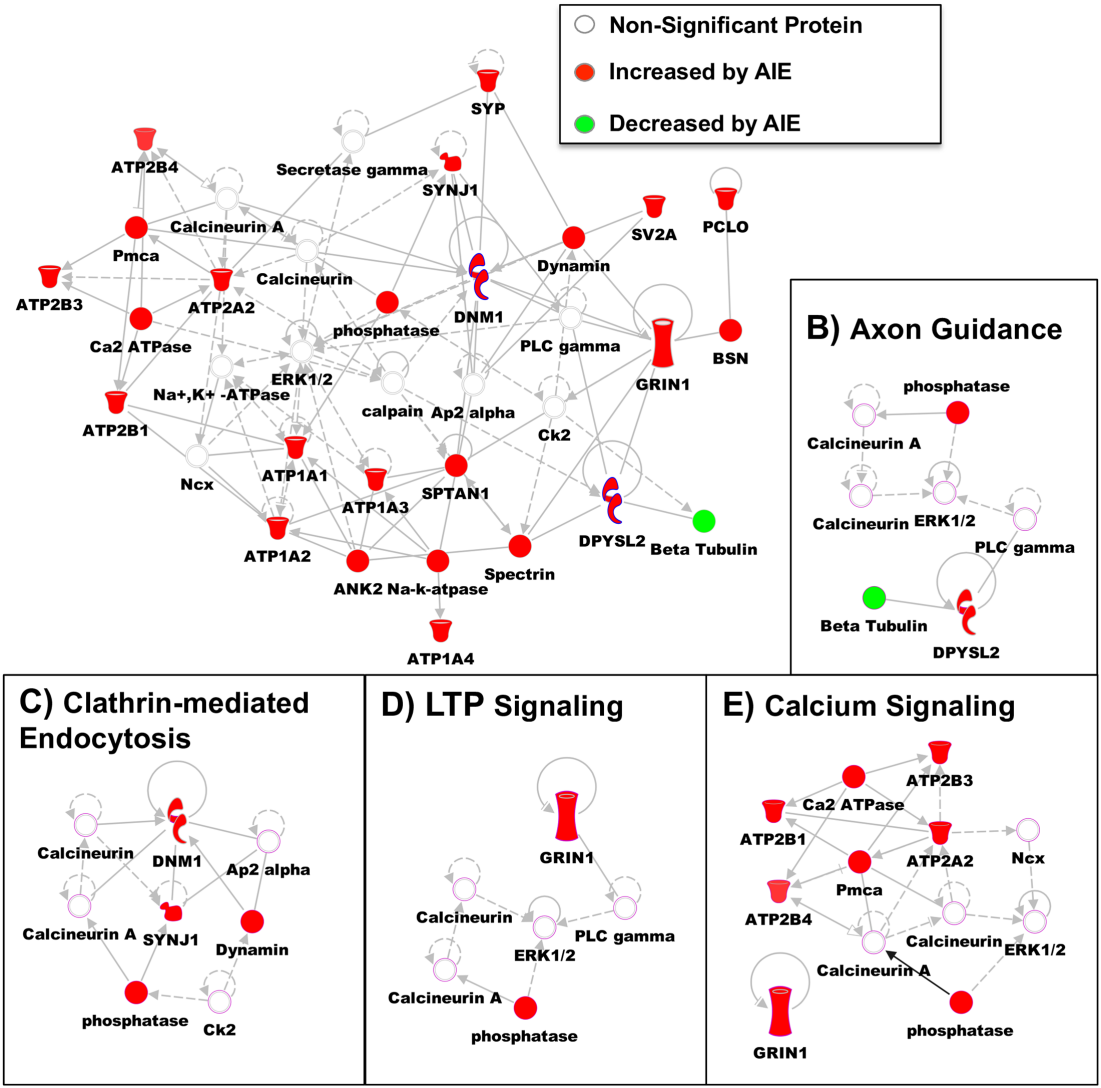
Pathway Analysis of GluN2B-associated Proteins Changed by AIE in the Non-Synaptic Fraction. (A) Modified diagram of protein interactions identified using Ingenuity pathway analysis (IPA). Analysis was performed on significantly changed non-synaptic/S2 proteins by AIE (see Table 2). Color represent if the protein was increased (red) by AIE or decreased (green) by AIE. Specific signaling pathways were also identified with their protein connections: (B) Axon Guidance, (C) Clathrin-mediated Endocytosis, (D) LTP Signaling, and (E) Calcium Signaling.

IPA was also used to identify disease and functional pathways that were significantly changed by AIE in the non-synaptic fraction. Table 4 provides a summary of the most physiologically relevant pathways that were significantly changed by AIE in the non-synaptic fraction: cell death and survival (apoptosis p = 3.49E-05, necrosis p = 3.77E-10, cell death p = 1.63E-07), cell morphology (neuritogenesis p = 1.90E-04, formation of cellular protrusions p = 3.79E-05), cell-to-cell signaling (neurotransmission p = 9.44E-06), cellular assembly and organization (microtubule dynamics p = 5.82E-09, organization of cytoskeleton p = 8.83E-10), cellular function and maintenance (cellular homeostasis p = 1.32E-04), cellular movement (cell movement p = 2.91E-03), molecular transport (transport of molecules p = 1.48E-08), nervous system development and function (development of neurons p = 5.10E-04, abnormal morphology of nervous system 1.53E-03, morphology of nervous system p = 2.21E-04), neurological disease (seizure disorder p = 9.54E-09, dyskinesia p = 5.29E-07, movement disorders p = 4.61E-13, schizophrenia p = 1.90E-06, tauopathy p = 1.22E-07, disorder of basal ganglia p = 1.32E-11, Huntington’s disease p = 1.50E-06), and nucleic acid metabolism (metabolism of nucleotide p = 1.35E-04). Network analysis was also provided for the significantly changed proteins in the non-synaptic fraction. One of the highly ranked networks was able to incorporate 18 of the proteins significantly altered by AIE (Fig 3A). The proteins in this network are involved in cellular function and maintenance, small molecule biochemistry, and molecular transport. Further, a few of the specific signaling pathways are highlighted in the insets: axon guidance (Fig 3B), clathrin-mediated endocytosis (Fig 3C), LTP signaling (Fig 3D), and calcium signaling (Fig 3E). It is also important to note that all but one of the proteins included in the assessed network were increased following AIE.

**Table 4:** Ingenuity Pathway Analysis of Significantly Changed S2 Proteins by AIE.

Categories	Diseases or Function	p-Value	Predicted Activation	Molecules	#
Cell Death and Survival	apoptosis	3.49E-05		ACO2, AGAP2, ALDOA, ATP1A1, ATP1A2, ATP2B4, C1QA, CANX, CCAR2, DLG4, DNM1, DSP, DYNC1H1, GLUD1, GNAQ, GRIN1, HK1, HSP90AA1, HSP90AB1, HSP90B1, HSPA9, PKM, SIRPA, SLC25A5, SYNGAP1, TUBA1A, UBA1	27
Cell Death and Survival	necrosis	3.77E-10		ACO2, AGAP2, ALDOA, ATP1A1, ATP1A2, ATP1A4, ATP2A2, ATP2B1, ATP2B4, C1QA, CCAR2, DLG4, DNM1, DSP, DYNC1H1, FGA, GLUD1, GNAQ, GRIN1, HK1, HSP90AA1, HSP90AB1, HSP90B1, HSPA9, PFKM, PKM, PLEC, SIRPA, SLC25A5, SYNGAP1, TUBA1A, TUBB, TUBB3, TUBB6, UBA1	35
Cell Death and Survival	cell death	1.63E-07		ACO2, AGAP2, ALDOA, ATP1A1, ATP1A2, ATP1A4, ATP2A2, ATP2B1, ATP2B4, C1QA, CANX, CCAR2, DLG4, DNM1, DSP, DYNC1H1, FGA, GLUD1, GNAQ, GRIN1, HK1, HSP90AA1, HSP90AB1, HSP90B1, HSPA9, PFKM, PKM, PLEC, SIRPA, SLC25A5, SYNGAP1, TUBA1A, TUBB, TUBB3, TUBB6, UBA1	36
Cell Morphology	neurito-genesis	1.90E-04		BSN, DLG4, DPYSL2, GRIN1, MAP6, Mbp, RIMS1, SIRPA, SV2A, SYNGAP1	10
Cell Morphology	formation of cellular protrusions	3.79E-05	Increased	BSN, CDH13, DLG4, DNM1, DPYSL2, GRIN1, HSP90AA1, MAP6, Mbp, PLEC, RIMS1, SIRPA, SV2A, SYNGAP1	14
Cell-To-Cell Signaling	neuro-transmission	9.44E-06		ATP1A2, BSN, DLG4, DNM1, GNAQ, GRIN1, RIMS1, SV2A, SYNGAP1, SYP	10
Cellular Assembly and Organization	microtubule dynamics	5.82E-09	Increased	ATP2B1, BSN, C1QA, CANX, CDH13, DLG4, DNM1, DPYSL2, DSP, GRIN1, HSP90AA1, MAP1A, MAP6, Mbp, PKM, PLEC, RIMS1, SIRPA, SV2A, SYNGAP1, TUBB, TUBB3	22
	organization of cytoskeleton	8.83E-10	Increased	ALDOA, ATP2B1, BSN, C1QA, CANX, CDH13, DLG4, DNM1, DPYSL2, DSP, GRIN1, HSP90AA1, MAP1A, MAP6, Mbp, PCLO, PKM, PLEC, RIMS1, SIRPA, SPTAN1, SV2A, SYNGAP1, TUBB, TUBB3	25
Cellular Function and Maintenance	cellular homeostasis	1.32E-04		ALDOA, ATP1A1, ATP1A2, ATP1A3, ATP1A4, ATP2A2, ATP2B3, ATP2B4, CKB, DLG4, GNAQ, GNB1, GRIN1, HSP90AA1, HSP90B1, PFKM, RIMS1, SV2A	18
Cellular Movement	cell movement	2.91E-03		AGAP2, ALDOA, ATP1A4, ATP2B4, CDH13, DNM1, DPYSL2, FGA, FGB, GNB1, GRIN1, HSP90AA1, HSP90AB1, HSP90B1, PKM, PLEC, SIRPA, TUBA1A, TUBB2B	19
Molecular Transport	transport of molecule	1.48E-08	Increased	ATP1A1, ATP1A2, ATP1A3, ATP1A4, ATP2A2, ATP2B1, ATP2B3, ATP2B4, ATP6V1A, CACNA2D1, CANX, DPYSL2, FGA, FGB, FGG, GNAQ, GOT2, GRIN1, HK1, HSPA9, RIMS1, SIRPA, SLC25A5, SV2A, SYNJ1	25
Nervous System Development and Function	abnormal morphology of nervous system	1.53E-03		ATP1A2, C1QA, CANX, CKB, DNM1, GRIN1, KRT1, PLEC, RIMS1, SYNGAP1	10
	morphology of nervous system	2.21E-04		ATP1A2, C1QA, CANX, CKB, DNM1, DPYSL2, GRIN1, KRT1, PLEC, RIMS1, SYNGAP1, TUBA1A	12
	development of neurons	5.10E-04		BSN, DLG4, DPYSL2, GRIN1, MAP6, Mbp, RIMS1, SIRPA, SV2A, SYNGAP1, SYP	11
Neurological Disease	seizure disorder	9.54E-09		ATP1A2, BSN, C1QA, C1QB, CACNA2D1, CKB, GNAQ, GRIN1, RIMS1, SPTAN1, SV2A, SYNGAP1, SYNJ1, TUBB	14
Neurological Disease	dyskinesia	5.29E-07		ATP2A2, ATP2B1, ATP5O, ATP6V1A, CKB, FGG, GNB1, GRIN1, HSP90AA1, PFKM, PKM, SV2A, SYNJ1, TUBA1A	14
Neurological Disease	Movement Disorders	4.61E-13	Decreased	ATP1A3, ATP2A2, ATP2B1, ATP2B3, ATP5O, ATP6V1A, CACNA2D1, CANX, CKB, FGG, GNAQ, GNB1, GRIN1, HSP90AA1, PFKM, PKM, SV2A, SYNGAP1, SYNJ1, TUBA1A, TUBA4A, TUBB2A, TUBB2B, TUBB3, TUBB4B, VPS35	26
Neurological Disease	Schizophrenia	1.90E-06		ATP1A1, ATP1A2, ATP1A3, ATP1A4, DLG4, DNM1, DPYSL2, GRIN1, MAP6,PCLO, SYP, VPS35	12
Neurological Disease	tauopathy	1.22E-07		CANX, DPYSL2, GRIN1, RIMS1, SV2A, SYNJ1, SYP, TUBA1A, TUBA4A, TUBB, TUBB2A, TUBB3, TUBB4B, VPS35	14
Neurological Disease	disorder of basal ganglia	1.32E-11		ATP1A3, ATP2A2, ATP2B1, ATP5O, ATP6V1A, CKB, FGG, GNB1, GRIN1, HSP90AA1, PFKM, PKM, SV2A, SYNJ1, TUBA1A, TUBA4A, TUBB2A, TUBB2B, TUBB3, TUBB4B, VPS35	21
Neurological Disease	Huntington's Disease	1.50E-06		ATP2A2, ATP2B1, ATP5O, ATP6V1A, CKB, FGG, GNB1, GRIN1, HSP90AA1, PFKM, PKM, SYNJ1, TUBA1A	13
Nucleic Acid Metabolism, Small Molecule Biochemistry	metabolism of nucleotide	1.35E-04		ALDOA, ATP1A1, ATP1A4, ATP2A2, ATP5O, GNAQ, GNB1, HSP90AA1, PKM,SLC25A5	10

## Discussion

This study finds that intermittent ethanol exposure during adolescence alters the GluN2B proteome in an enduring way that persists into adulthood. The persistence of the changes is particularly noteworthy because they are apparent 20–25 days after the last ethanol exposure. These long-term adaptations in GluN2B signaling could be of mechanistic significance for the hippocampal phenotypes that are associated with AIE (e.g. memory deficits, lowered LTP threshold, and increase in immature spines [3, 32]), since many of the pathways identified are functionally relevant to these phenotypes. For example, we found that significantly altered synaptic proteins were most highly represented in pathways responsible for formation of cellular protrusions, organization of the cytoskeleton, and cell proliferation. These signaling pathways could contribute to the changes in dendritic morphology [3] or the apparent loss of neurons [33] that are observed in adulthood after AIE.

A similar GluN2B targeted proteomic approach was performed following chronic intermittent ethanol exposure and withdrawal in adult mice [27]. Similar to the previous study, the synaptic fraction was highly enriched for PSD-95 and other PSD scaffolding proteins illustrating the effective separation of synaptic and non-synaptic proteins. Further, this earlier work provided an inventory of proteins that seem to have non-specific interactions with the GluN2B antibody. This was determined through GluN2B-IP proteomic screen in tissue from GluN2B knock out (KO) mice [27]. This KO tissue eliminated 105 non-specific proteins. These non-specific proteins were compared to the current lists of significantly altered proteins (Tables 1 & 2). Of all of these altered proteins, only 4 proteins were found on this KO list (S2: regulating synaptic membrane exocytosis protein 1, fibrinogen gamma chain, complement C1q subcomponent subunit A and, aspartate aminotransferase mitochondrial) demonstrating the specificity of the remaining 103 proteins.

In this adult study, there were 829 proteins identified across treatments and subcellular fractions with 64 significantly changed by chronic ethanol in the synaptic and 22 changed in the non-synaptic. In the current study, we identified 693 proteins and found 34 proteins changed by AIE in the synaptic fraction and 73 proteins changed in the non-synaptic fraction. This comparison demonstrates that size of the GluN2B proteome was similar between experiments but that ethanol uniquely regulated these proteins in different subcellular compartments. This difference could be a result of many factors: age of ethanol administration, time of tissue collection (5hrs versus 25 days after ethanol exposure), species (mouse versus rat), and route of ethanol administration (vapor versus intragastric). Aside for the exact cause, the preferential enhancement of non-synaptic proteins suggests that extrasynaptic GluN2B NMDAR signaling may be more profoundly altered in the current study.

### Adolescent Intermittent Ethanol Alters Cytoskeletal Arrangement

In the synaptic fraction, several members of the alpha actinin family (1, 2, and 4) showed increased association with GluN2B following AIE. The alpha actinin families of proteins are known to link GluN2B-containing NMDARs to the actin cytoskeleton. One of the functions of this interaction is to target CaMKII to GluN2B-NMDARs and in the case of alpha-actinin 2 to do this in a Ca^2+^-independent manner [34]. The function of this increase in targeting has been shown to alter dendritic morphology, where overexpression of alpha actinin 2 produced increased dendritic length and number of protrusions in hippocampal neurons [35] along with a disorganized postsynaptic density. This work suggests that this dendritic phenotype could arise from overly linked actin filaments. Thus, this increase in alpha actinin association with GluN2B-NMDARs could provide a molecular basis for the increase in immature spines seen following AIE in our earlier work [3].

In addition to the AIE effects on dendritic spine morphology, we also found that signaling involved in axon formation and integrity was dynamically regulated. One group of proteins altered by AIE that are associated with this signaling are spectrin proteins (spectrin alpha 1,spectrin beta 1 and 2) and their adapter protein, Ankyrin-2. Spectrins are involved in structural stability, polarity, and trafficking of proteins in the axon. Ankyrin-2 mediates the interactions of proteins, such as ion pumps and channels, to the spectrin cytoskeleton. Disruption of this Ankyrin 2-spectrin interaction could lead to loss of axonal integrity. Both preclinical and clinical studies have found that one of the long-term consequences of adolescent alcohol exposure is decrease in white matter, which could result from changes in axonal integrity [36, 37].

### Adolescent Intermittent Ethanol-Induced Changes in Calcium Signaling

Pathway analysis also demonstrated that there was independent regulation of calcium signaling between the two fractions. In the synaptic fraction there was an overall decrease in association of GluN2B with CaMKIIβ and F-actin, as well as actin and ATPases. In contrast, in the non-synaptic fraction there was an increase in association of GluN2B with proteins involved in calcium signaling (Ca^2+^ transporting ATPases, Sodium/potassium-transporting ATPase subunit). This signaling pathway is important because of the role of CaMKII in mediating synaptic plasticity (e.g. LTP) in the hippocampus. We found that the CaMKIIβ isoform was decreased in its association with GluN2B in the synaptic fraction. CaMKIIαaMKIIecreasefrom cytosolic regions to dendritic spines in CA1 pyramidal neurons through binding of CaMKIIβaMKIIytosol under basal conditions [38]. A decreased association of CaMKIIβaMKIIased associatireflect a transition of CaMKIIαaassociation with GluN2B rather than associating in its inactive form with F-actin. The activation of CaMKIIα and dissociation from F-actin has also recently been shown to allow for F-actin remodeling and stabilization of the dendritic spine [39]. Therefore, these alterations in CAMKII and F-actin by AIE could help explain the changes in LTP threshold and dendritic morphology found in earlier AIE studies [3].

### Adolescent Intermittent Ethanol-Induced Trafficking of GluN2B-NMDARs

During chronic ethanol exposure there is an increase in synaptic clustering of GluN2B-NMDARs [40, 41]. Following this enhancement of GluN2B at the synapse, these receptors move to extrasynaptic locations during ethanol withdrawal [26]. We have reported that during ethanol withdrawal in adult mice there is a dissociation of GluN2B from PSD scaffolding proteins, which likely promotes this re-localization of GluN2B signaling to extrasynaptic locations [27]. The present findings show that there are a greater number of significantly changed proteins in the non-synaptic compared to the synaptic fraction following AIE. While there was no detectable change in GluN2B levels with AIE, there is an increase in GluN1 subunit association in this non-synaptic fraction. The NMDAR is heterotetrameric complex that contains two GluN1 subunits and two either GluN2 or GluN3 subunits [13]. The GluN2A and GluN2B subunits are the primary subunits in the mature hippocampus and the primary subunits detected in this proteomic screen. These subunits can form di- (two GluN2A or two GluN2B) or tri- heterotetrameric complexes (one GluN2B and one GluN2A). Thus this enhanced GluN1 association following AIE could represent a shift from tri-heterotetrameric receptors to di-heterotetrameric receptors. This shift in subunit composition would alter the NMDAR transmission as well as intracellular signaling cascades. The data presented here does suggest that non-synaptic GluN2B associated signaling may be altered by AIE. These long-term alterations in non-synaptic signaling could cause the hippocampal synapse to be differentially sensitive to alcohol exposures in adulthood, and/or could predispose hippocampal neurons to excitotoxicity associated with excessive activation of excitatory extrasynaptic receptors. Extrasynaptic GluN2B transmission is involved in various pathological conditions [e.g. Huntington’s disease, ischemia, Parkinson’s disease, Alzheimer's disease [42, 43] and it could be this aberrant signaling that causes the prolonged effects from AIE.

### Conclusion

In this study, we identified a number of proteins whose association with the GluN2B subunit was significantly altered by AIE in adulthood. These proteins may provide the molecular basis for the changes in spine morphology, LTP thresholds, and ethanol-induced spatial memory impairment produced by AIE. While this study has identified many potentially important proteins, further studies using western blot analysis and electrophysiology are needed to validate these proteomic changes and determine what functional role they may have. Additionally, this proteomic screen was able to identify a large number of proteins but it is possible that additional proteins could be detected with a larger sample size. Further, these results show that these protein changes are present at P70, which is considered early adulthood in the rodent. Future work is needed to determine the longevity of these protein changes and how they relate to hippocampal physiology and behavior.

## Supporting Information

S1 Dataset Complete Proteomic Dataset. Proteins and accession numbers were identified using NIST rat ion trap library. The next groups of columns provide spectral counts for all samples (n = 4) from adolescent intermittent ethanol exposure (AIE) or adolescent intermittent saline exposure (AIS) in the synaptic and non-synaptic fractions.
(XLSX)
